# Impact of socioeconomic factors on lifestyle changes among employees of public higher education institutions in ELSA-Brasil during COVID-19 pandemic

**DOI:** 10.1590/0102-311XEN047123

**Published:** 2023-11-13

**Authors:** Maria del Carmen Bisi Molina, Carla Moronari de Oliveira Aprelini, Adriana Lúcia Meireles, Rosane Harter Griep, Luana Giatti, Maria da Conceição Chagas de Almeida, Maria de Jesus Mendes da Fonseca, Maria Inês Schmidt, Sandhi Maria Barreto, Sheila Maria Alvim de Matos, Alvaro Vigo, José Geraldo Mill

**Affiliations:** 1 Centro de Ciências da Saúde, Universidade Federal do Espírito Santo, Vitória, Brasil.; 2 Programa de Pós-graduação em Saúde e Nutrição, Universidade Federal de Ouro Preto, Ouro Preto, Brasil.; 3 Instituto Oswaldo Cruz, Fundação Oswaldo Cruz, Rio de Janeiro, Brasil.; 4 Faculdade de Medicina, Universidade Federal de Minas Gerais, Belo Horizonte, Brasil.; 5 Hospital das Clínicas, Universidade Federal de Minas Gerais, Belo Horizonte, Brasil.; 6 Instituto Gonçalo Moniz, Fundação Oswaldo Cruz, Salvador, Brasil.; 7 Escola Nacional de Saúde Pública Sergio Arouca, Fundação Oswaldo Cruz, Rio de Janeiro, Brasil.; 8 Hospital de Clínicas de Porto Alegre, Universidade Federal do Rio Grande do Sul, Porto Alegre, Brasil.; 9 Instituto de Saúde Coletiva, Universidade Federal da Bahia, Salvador, Brasil.; 10 Programa de Pós-graduação em Epidemiologia, Universidade Federal do Rio Grande do Sul, Porto Alegre, Brasil.; 11 Departamento de Ciências Fisiológicas, Universidade Federal do Espírito Santo, Vitória, Brasil.

**Keywords:** COVID-19 Pandemic, Diet, Life Style, Risk Factors, Pandemia COVID-19, Dieta, Estilo de Vida, Fatores de Risco, Pandemia de COVID-19, Dieta, Estilo de Vida, Factores de Riesgo

## Abstract

This study aimed to identify lifestyle changes and associated sociodemographic factors in women and men participating in the *Brazilian Longitudinal Study for Adult Health* (ELSA-Brasil) cohort during the COVID-19 pandemic. Longitudinal study with 3,776 (aged 58.8 years; SD ± 8.5) employees of public higher education institutions in the second follow-up and the wave-COVID of ELSA-Brasil. Data collected using structured questionnaires. An exploratory analysis was performed using binary and multinomial logistic regression on the dependent variables with two and three categories, respectively, by obtaining crude and adjusted odds ratio estimates in SPSS 20.0, considering a p-value < 0.05. There was a reduction in physical activity of 195.5 (SD ± 1,146.4) metabolic equivalents per week in women and 240.5 (SD ± 1,474.2) in men, and in smoking by 15.2%. There was an increase in alcohol consumption in men and women (434.2 ± 5,144.0; and 366.1 ± 4,879.0, respectively), in the food quality score (0.8 ± 3.7, women; 0.5 ± 3.7, men), sleeping time (0.4 ± 1.2, women; 0.5 ± 1.1, men), screen time (1.7 ± 2.4, women; 1.4 ± 2.3, men), and sitting time (1.7 ± 2.6, women; 1.5 ± 2.4, men) (hours/day). In total, 18.6% increased the purchase of ultra-processed foods and 36% increased the purchase of natural foods. Age and work activity contributed to increase the chance of purchasing ultra-processed foods, and age and adherence to social distancing influenced the shift to a more sedentary behavior, while income and active work favored the increase in alcoholic beverage consumption. These factors should be considered when developing public policies to avoid individual behaviors that are harmful to health during pandemics.

## Resumo

O objetivo do estudo é identificar mudanças no estilo de vida e fatores sociodemográficos associados em mulheres e homens participantes da coorte Estudo Longitudinal da Saúde do Adulto (ELSA-Brasil) durante a pandemia de COVID-19. Estudo longitudinal com 3.776 (58,8 anos; DP ± 8,5) funcionários de instituições públicas de Ensino Superior no segundo acompanhamento e na onda COVID do ELSA-Brasil. Os dados foram coletados por meio de questionários estruturados. Foi realizada análise exploratória por meio de regressão logística binária e multinomial nas variáveis dependentes com duas e três categorias, respectivamente, obtendo-se estimativas brutas e ajustadas de *odds ratio* no SPSS 20.0, considerando um valor de p < 0,05. Houve redução da atividade física de 195,5 (DP ± 1.146,4) equivalentes metabólicos por semana nas mulheres e de 240,5 (DP ± 1.474,2) nos homens, e do tabagismo de 15,2%. Houve aumento do consumo de álcool em homens e mulheres (434,2 ± 5.144,0 e 366,1 ± 4.879,0, respectivamente), do escore de qualidade alimentar (0,8 ± 3,7, mulheres; 0,5 ± 3,7, homens), do tempo de sono (0,4 ± 1,2, mulheres; 0,5 ± 1,1, homens), do tempo de tela (1,7 ± 2,4, mulheres; 1,4 ± 2,3, homens) e do tempo sentado (1,7 ± 2,6, mulheres; 1,5 ± 2,4, homens) (horas/dia). Além disso, 18,6% aumentaram a compra de alimentos ultraprocessados e 36% aumentaram a compra de alimentos naturais. A idade e a atividade laboral contribuíram para aumentar a chance de compra de alimentos ultraprocessados, e a idade e a adesão ao distanciamento social influenciaram a mudança para um comportamento mais sedentário, enquanto a renda e o trabalho ativo favoreceram o aumento do consumo de bebidas alcoólicas. Estes fatores devem ser considerados na elaboração de políticas públicas a fim de evitar comportamentos individuais deletérios à saúde em períodos de pandemia.

## Resumen

El objetivo de este estudio es identificar los cambios en el estilo de vida y los factores sociodemográficos asociados en mujeres y hombres que participan en la cohorte *Estudio Longitudinal de Salud del Adulto en Brasil* (ELSA-Brasil) durante la pandemia de la COVID-19. Estudio longitudinal con 3.776 (58,8 años; DE ± 8,5) funcionarios en instituciones públicas de educación superior en el segundo seguimiento y en la ola COVID de ELSA-Brasil. Los datos se recopilaron de cuestionarios estructurados. El análisis exploratorio se realizó mediante regresión logística binaria y multinomial en variables dependientes con dos y tres categorías, respectivamente, en la cual se obtuvieron estimaciones brutas y ajustadas de *odds ratios* en SPSS 20.0, teniendo en cuenta un valor de p < 0,05. Hubo una reducción en la actividad física de 195,5 (DE ± 1.146,4) equivalentes metabólicos por semana en mujeres y de 240,5 (DE ± 1.474,2) en hombres, y del tabaquismo del 15,2%. Hubo un aumento en el consumo de alcohol en hombres y mujeres (434,2 ± 5.144,0 y 366,1 ± 4.879,0, respectivamente), en el puntaje de calidad de los alimentos (0,8 ± 3,7, mujeres; 0,5 ± 3,7, hombres), en el tiempo de sueño (0,4 ± 1,2, mujeres; 0,5 ± 1,1, hombres), en el tiempo frente a la pantalla (1,7 ± 2,4, mujeres; 1,4 ± 2,3, hombres) y en el tiempo sentado (1,7 ± 2,6, mujeres; 1,5 ± 2,4, hombres) (horas/día). Además, el 18,6% aumentó la compra de alimentos ultraprocesados y el 36% la compra de alimentos.

## Introduction

The spread of COVID-19 significantly affected populations worldwide. It was not only associated with disease and high mortality, but also with several lifestyle changes, such as diet, smoking, physical activity, alcohol consumption, sitting time, sleep, among others, being found in all countries, regardless of the progression, control, or prevention of the disease ^1^ ^,^ ^2^.

In Brazil, some studies have demonstrated that these changes occurred in different locations ^3^ ^,^ ^4^ ^,^ ^5^ with repercussions on the cardiovascular health and the immune system, substantially impacting health and the response to infections ^6^ ^,^ ^7^. Furthermore, negative changes have had an impact on mental health, being related to symptoms of anxiety, stress, and depression ^8^.

Some studies found a worsening of lifestyle habits and an increase in health risk behaviors during the period of social restrictions ^1^ ^,^ ^4^ ^,^ ^8^ ^,^ ^9^. A review showed that lockdown affected the dietary practices of various populations, with potential short- and long-term effects on global health ^10^, and several studies identified the negative effect of the pandemic on physical activity and sedentary behavior ^8^. However, there are no studies that indicate the factors associated with such changes during this period in Brazil. In other parts of the globe, studies have indicated an association between unhealthy behaviors and racial/ethnic minority groups, younger individuals, lower education, and gender ^11^ ^,^ ^12^ ^,^ ^13^.

Therefore, it is necessary to understand lifestyle changes resulting from a pandemic and their associated factors to guide public policies to improve the resilience and effectiveness of health approaches during epidemics. Thus, this study aims to identify changes in lifestyle and associated sociodemographic factors in women and men participating in the *Brazilian Longitudinal Study of Adult Health* (ELSA-Brasil) cohort during the COVID-19 pandemic.

## Methods

### Study population

ELSA-Brasil is a multicenter cohort study with 15,105 active or retired civil servants (aged 35 to 74 years) at baseline, from higher education and research institutions located in six Brazilian capitals: Belo Horizonte (Minas Gerais State), Porto Alegre (Rio Grande do Sul State), Rio de Janeiro, São Paulo, Vitória (Espírito Santo State), and Salvador (Bahia State) ^14^. Face-to-face monitoring of the participants was carried out at three moments: baseline (2008-2010), first follow-up (2012-2014) and second follow-up (2017-2019). At all stages, data were collected in a standardized way via structured questionnaires, with protocols and trained staff, preceded by pilot studies, in addition to other strategies to ensure data quality ^15^ ^,^ ^16^.

Following the same quality rigor, from July 2020 to February 2021, a complementary study by ELSA-Brasil (titled wave-COVID) was carried out with the objective of evaluating the short- and medium-term impact of COVID-19. For this study, five research centers participated in the survey. The São Paulo research centers did not participate because it had initiated a separate survey at the time that considered similar questions. Participants were invited to answer the questionnaires digitally, using an application produced specifically for the study, with the help of a trained team. Four questionnaire modules were included, covering adherence to social distancing, exposure, signs and symptoms of COVID-19, lifestyle habits, diet, among others. Participants with difficulties accessing the application were given the option of a telephone interview.

**Figure 1:**
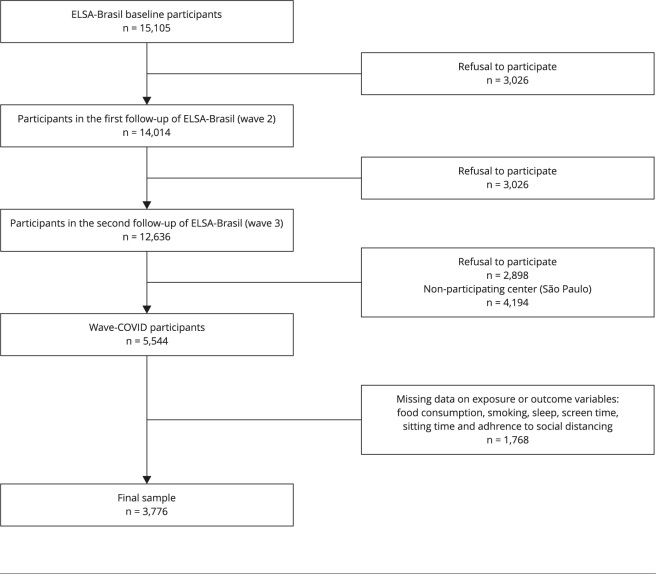
Flowchart of study participants.

The study analyses were carried out using the second follow-up database, consisting of 12,636 individuals, and 5,544 participants of wave-COVID. Individuals who did not participate in both stages (n = 7,092); and without information on exposure and/or outcome variables (n = 1,768), totaling 3,776 participants (Figure 1).

### Study variables

### Exposure variables

The wave 3 variables were used: age ranged from 42 to 82 years, categorized as: under 59/over 60, sex (female; male), skin color (white; black/mixed-race/indigenous), education (incomplete/complete primary education; complete secondary education; complete higher education), per capita family income (tertile/USD), occupational status (active; retired), marital status (married/living together; separated/single/widowed/other), physical activity, diet quality score, alcohol consumption, smoking and average sleep time. The following information from wave-COVID was considered: adherence to distancing, physical activity, diet, alcohol consumption, smoking, average sleep time, change in food purchases during the pandemic, screen time, and sitting time.

To identify adherence to social distancing, the following question was considered: “Are you following the recommendations to stay/work at home and go out ONLY to make essential health-related purchases?”. With answer options: “Yes, totally”; “Yes, partially, because I go out regularly to take care of a family member/person outside the home”; “No, because I continue working”; “No, because I don’t agree with the measures”; and “No, for another reason”. The first two answers were considered affirmative to distancing.

### Outcome variables - life habits

### a) Physical activity

Leisure-time physical activity, measured by the *International Physical Activity Questionnaire* (IPAQ) ^17^ was recorded in minutes/week using the product of the weekly frequency and duration of each of the activities performed - walking, moderate, and vigorous. Vigorous activities were classified as those that require great physical effort and make you breathe much faster than normal; and medium/moderate as those that require medium physical effort and that make you breathe a little faster than normal. Activities lasting at least 10 minutes in a row, carried out during leisure time, for physical conditioning, recreation, or sport were also considered. Domestic chores and commuting were not considered.

In the wave-COVID, the questions considered were: “Since the beginning of social distancing, on average, how many days per week do you go walking in your free time?”, “Since the beginning of social distancing, how many days per week do you go walking? Do you practice vigorous physical activities in your free time?”, and “Since the beginning of social distancing, how many days a week do you practice medium/moderate physical activities?”. The duration of the activities was also questioned ^18^. The calculation was performed in the same way.

Subsequently, the data were converted into multiples of metabolic equivalents per week, for each intensity, considering the IPAQ presented:

Walking = 3.3 × frequency (days/week) × duration (minutes/day);

Moderate = 4 × frequency (days/week) × duration (minutes/day);

Vigorous = 8 × frequency (days/week) × duration (minutes/day).

The total sum of metabolic equivalents was used as continuous.

Individuals who do not engage in physical activity received a score of zero in the analysis, given the possibility of modifying their habits later.

### b) Food quality score

To assess the diet quality, the *Food Frequency Questionnaire* (FFQ) was developed and validated for ELSA-Brasil ^18^. For wave 3, a semi-quantitative FFQ with 76 items was developed. In the wave-COVID, the items from wave 3 were organized into 22 food groups, to reduce the application time of the instrument, and only the frequency of consumption was considered ^19^.

The *Food Quality Score* (FQS) was developed considering the 22 items and the 5 consumption frequency answer options present in the wave-COVID (“1 or more times a day”, “5 to 6 times per week”, “1 to 4 times per week”, “1 to 3 times per month”, and “never/almost never”). Briefly, each food or food group was assigned a score (+1 or -1) according to the frequency of healthy and unhealthy food consumption markers ^20^ ^,^ ^21^, which resulted in a minimum score of -15 and a maximum of +15 (Supplementary Material: Supplementary Material https://cadernos.ensp.fiocruz.br/static//arquivo/suppl-e00047123-ing_4876.pdf), with the maximum score referring to the highest diet quality.

### c) Change in food purchase

To assess whether there was a change in the purchase of food during the COVID-19 pandemic, the question considered was: “During social distancing, was there a change in relation to the purchase of food for your home?”, with the answer options: “No”, “Yes, more convenience foods, such as lasagna, hamburgers, nuggets, etc.”, “Yes, more fresh foods were purchased”, “Yes, they were industrialized foods, such as sausages, canned goods, cookies, cakes, soft drinks”. Therefore, a self-perceived change in food acquisition was reported.

For the change in food purchase, three categories were considered: No; Yes, with more fresh foods; and Yes, with more ultra-processed foods (a mixture of convenience and industrialized foods).

Those who reported greater purchase of natural foods and/or ultra-processed foods in the wave-COVID were categorized as “increased habit”; and as “maintained habit” for those who did not report buying more of these foods.

### d) Alcohol consumption

Alcohol consumption (mL/week) was estimated using the FFQ, with data on frequency and quantity of beer, wine and spirits. To identify the quantity, the variables were converted into times per week and then multiplied by the quantity consumed each time (mL), the sum of alcoholic beverage consumption was used to obtain the total intake in mL/week ^18^ ^,^ ^19^.

### e) Smoking

Individuals who had smoked more than 100 cigarettes in their lifetime and continue smoking were classified as smokers; former-smoker, those who have smoked 100 cigarettes and stopped; and non-smokers were those who smoked less than 100 cigarettes. Smokers and non-smokers (former-smokers and those who never smoked) were considered in the two collection stages.

Those who did not smoke cigarettes in wave 3 and started smoking in wave-COVID were categorized as “increased habit”; those who smoked cigarettes and stopped doing so, as “decreased habit”; and those who did not change their habit, as “maintained habit”.

### f) Average sleep time 

To assess the participants’ sleep time, the average sleep time in hours reported ^20^ in the questionnaires applied before and during the COVID-19 pandemic was considered, by the question: How many hours on average do you sleep in a normal night’s sleep?

### g) Screen time

Self-perceived changes in screen time were identified by the questions in the wave-COVID: “On a typical day, before the start of social distancing, how much time did you spend in front of any screen (smartphone, computer, TV, notebook, or others)?” and “How much time do you now spend in front of any screen?” (hours/day) as presented in the long version of IPAQ, validated for Brazil ^21^.

### h) Sitting time

To estimate sitting time (hours/day), self-perceived changes in the wave-COVID were also identified: “On average, how much time did you spend sitting, reclining or lying down daily, before social distancing?” and “Since social distancing, how much time have you spent sitting, reclining or lying down daily?”. The questions excluded sleeping hours.

### Statistical analysis

A descriptive analysis of the study population was carried out, stratified by sex, according to sociodemographic variables and lifestyle habits, using absolute and relative frequencies, with the chi-square test.

As small changes in continuous variables are sensitive, the assessment of changes in habits during the pandemic was categorized in the same way as Xu et al. ^22^ did for diet quality. However, we did the same for all outcome variables and adapted the categorization without considering the scale of decreasing or increasing changes: (i) decrease in habit when < -3%; (ii) habit maintained when ≥ -3% and ≤ -3%; and (iii) increased habit when > +3%.

To identify the factors associated with changes in habits, exploratory analysis was performed using multinomial logistic regression on the dependent variables with three categories (decreased, maintained, or increased) and binary logistic regression on the dichotomous dependent variables (maintained or increased). Associations between changes in lifestyle habits and independent variables were verified by obtaining crude and adjusted estimates of the odds ratios (OR), using 95% confidence intervals (95%CI). Smoking was not included in the regression due to the small number of individuals who changed this habit (n = 65 decreased; n = 24 increased).

For the adjusted analyses, variables that presented p < 0.20 in the crude analyses were included, following the order of a hierarchical model to determine the outcomes (socioeconomic, occupational, marital, and behavioral variables - adherence to social distancing).

Data analyses were done using SPSS IBM Statistics program version 20.0 (https://www.ibm.com/), considering p-value < 0.05 as statistically significant.

All stages of ELSA-Brasil were approved by the Research Ethics Committees of the institutions involved [Acts 343/06 (Oswaldo Cruz Foundation - Fiocruz), 041/06 (Federal University of Espírito Santo - UFES), 186/06 (Federal University of Minas Gerais - UFMG), 194/06 (Federal University of Rio Grande do Sul - UFRGS), 027/06 (Federal University of Bahia - UFBA). And wave-COVID was approved by: CAAE: 32778620.1.0000.5030/4.067.18 (UFBA); CAAE: 56021516.0.1001.5240/4.063.982 (Fiocruz); CAAE: 32061620.5.0000.5060/4.090.940 (UFES); CAAE: 48608515.5.1001.5327/4.023.601 (UFRGS); CAAE: 47125015.4.1001.5149/4.082.055 (UFMG)]. Before starting collections, the participants signed an informed consent form.

## Results

Sample consisted of 3,776 participants (58.4% women), 55% individuals aged up to 59 years, 58.7% White skin color, 35.1% with an average per capita income of USD 478.0-868.0, 69.4% were more educated, 73.9% active workers, 63.3% married, and 84.5% adhered to social distancing. Women had higher levels of education and represented 67.3% of retirees, 78.3% of those with a “separated/single/widowed/other” marital status, and 59.2% of those who adhered to social distancing (p < 0.05) (Table 1).

**Table 1:** Socioeconomic characteristics of study participants according to sex (n = 3,776). *Brazilian Longitudinal Study of Adult Health* (ELSA-Brasil), 2017-2021.

Characteristics	Female (n = 2,207)		Male (n = 1,569)		p-value
	n	%	n	%	
Age (years)					0.163
40-59	1,235	59.5	842	40.5	
Over 60	972	57.2	727	42.8	
Skin color (n = 3,741) *					0.263
White	1,266	57.5	926	42.3	
Black/Mixed-race/Indigenous	920	59.5	620	40.5	
Per capita income in USD (n = 3,770) *					0.714
< 477.0	728	58.0	527	42.0	
478.0-868.0	767	58.0	555	42.0	
> 869.0	709	59.4	484	40.6	
Education					< 0.001
Incomplete/Complete primary education	75	39.7	114	60.3	
Complete secondary education	606	62.6	362	37.4	
Complete higher education	1,526	58.3	1,093	41.7	
Occupational situation (n = 3,773) *					< 0.001
Active	1,542	55.3	1,246	44.7	
Retired	663	67.3	322	32.7	
Marital status					< 0.001
Married/Living together	1,122	46.9	1,268	53.1	
Separated/Single/Widowed/Other	1,085	78.3	301	21.7	
Adherence to social distancing **					0.035
No	320	54.5	267	45.5	
Yes	1,887	59.2	1,302	40.8	

Note: chi-square test.

* n different due to sample losses;

** Wave-COVID information ^28^.

There were significant changes during the pandemic in physical activity, with a reduction of 195.5 (standard deviation - SD ± 1,146.4) and 240.5 (SD ± 1,474.2) metabolic equivalents/week in women and men, respectively; a 15.2% (p < 0.001) reduction in smoking and an increase in alcohol consumption (mL/week) in men and women (434.2 SD ± 5,144.0; and 366.1 SD ± 4,879.0, respectively) (Table 2).

**Table 2:** Lifestyles before and during the COVID-19 pandemic according to gender (n = 3,776). *Brazilian Longitudinal Study of Adult Health* (ELSA-Brasil), 2017-2021.

Characteristics	Before		During		Change		
Female						
Physical activity (metabolic equivalents/week)	808.6	± 1,070.8	613.1	± 988.2	-195.5 *	± 1,146.4
FQS	4.8	± 3.7	5.5	± 3.9	0.8 *	± 3.7
Alcoholic beverage (mL/week)	513.5	± 1,077.0	879.7	± 4,979.4	366.1 *	± 4,879.0
Average sleep time (hours/day)	6.6	± 1.3	7.0	± 1.2	0.4 *	± 1.2
Screen time (hours/day)	5.0	0 ± 2.9	6.7	± 3.6	1.7 *	± 2.4
Sitting time (hours/day)	5.5	± 3.6	7.2	± 4.2	1.7 *	± 2.6
Smoker [n (%)]	151	(6.8)	129	(5.8)	-22 *	(-14.6)
Male						
Physical activity (metabolic equivalentss/week)	1,165.7	± 1,427.4	925.2	± 1,224.4	-240.5 *	± 1,474.2
FQS	4.2	± 3.6	4.8	± 3.8	0.5 *	± 3.7
Alcoholic beverage (mL/week)	1,748.0	± 3,560.1	2,182.3	± 5,584.2	434.2 **	± 5,144.0
Average sleep time (hours/day)	6.5	± 1.2	6.9	± 1.3	0.5 *	± 1.1
Screen time (hours/day)	5.4	± 3.1	6.8	± 3.6	1.4 *	± 2.3
Sitting time (hours/day)	6.2	± 3.7	7.7	± 4.3	1.5 *	± 2.4
Smoker [n (%)]	118	(7.5)	99	(6.3)	-19 *	(-16.1)

FQS: *Food Quality Score*; SD: standard deviation.

Note: values are mean ± SD or n (%). Paired t-test.

* p-value < 0.001.

** p-value < 0.05.

The FQS showed an increase of 0.8 (SD ± 3.7) points among women and 0.5 (SD ± 3.7) points among men (p < 0.001). There was also an increase, in both genders, in sleep time (0.4 ± 1.2, women; 0.5 ± 1.1, men), screen time (1.7 ± 2.4, women; 1.4 ± 2.3, men), and SiT (1.7 ± 2.6, women; 1.5 ± 2.4, men) (hours/day) (p < 0.001). Regarding food acquisition, 18.6% of participants increased their purchase of ultra-processed foods (11.2% and 7.4% among women and men, respectively), and 36% increased their purchase of fresh foods (23% women and 13% men) (descriptive analysis, data not shown in the table).

**Figure 2:**
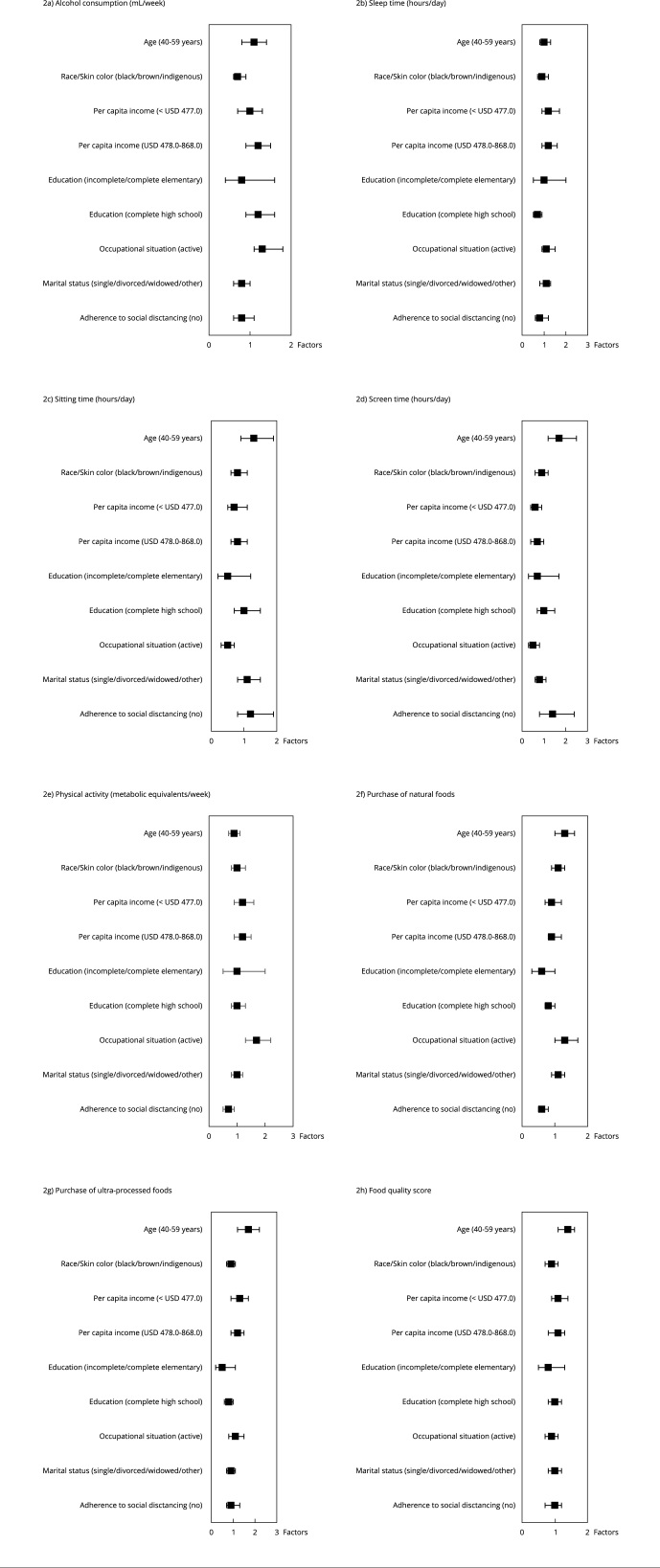
Factors associated with changes in lifestyle habits during the COVID-19 pandemic among women. *Brazilian Longitudinal Study of Adult Health* (ELSA-Brasil), 2017-2021.

Note: multinomial logistic regression on the dependent variables with three categories − reference to the first (decreased), category not shown. Binary logistic regression on dichotomous dependent variables. Final model adjusted for age, skin color, per capita income, educational level, occupational status, marital status and adherence to social distancing when p < 0.2 in the crude analysis. Data are odds ratios (95% confidence intervals). * p-value < 0.05; ** p-value < 0.001.

Socioeconomic, occupational and behavioral factors were analyzed regarding changes in individuals’ lifestyle habits. Among the factors associated with changes in women’s habits, younger participants (40-59 years) had 1.4 times (95%CI: 1.1-1.6; p = 0.001) the chance of increasing FQS, but also 1.7 (95%CI: 1.2-2.2; p < 0.001) and 1.3 (95%CI: 1.0-1.6; p = 0.010) times the chance of increasing the purchase of ultra-processed foods and fresh foods, respectively. Furthermore, women were 70% (95%CI: 1.2-2.5; p = 0.003) more likely to have increased screen time (Figure 2).

Women of “black/mixed-race/indigenous” skin color were 30% less likely to increase alcohol consumption (95%CI: 0.6-0.9; p = 0.003). Those in the lowest tertile of per capita income were 40.0% less likely to increase their screen time (95%CI: 0.4-0.9; p = 0.030). Regarding education, having incomplete/complete primary and secondary education decreased the chances by 40% (95%CI: 0.3-1.0; p = 0.040) and 20% (95%CI: 0.7-1.0; p = 0.040), respectively, to increase the purchase of fresh foods, and women with complete secondary education were 30% less likely to increase their sleep time (95%CI: 0.5-0.9; p = 0.040).

Active female workers were 1.3 (95%CI: 1.1-1.8; p = 0.040) times more likely to increase alcohol consumption, but also more likely to increase their purchase of fresh foods (1.3; 95%CI: 1.0-1.7; p = 0.020) and physical activity (1.7; 95%CI: 1.3-2.2; p < 0.001), and 50% less likely to increase the screen time and sitting time (95%CI: 0.3-0.8; p = 0.002, 95%CI: 0.3-0.7; p = 0.001, respectively). Marital status did not interfere in changing women’s habits. However, those who did not adhere to social distancing presented 40% (95%CI: 0.5-0.8; p = 0.001) and 30% (95%CI: 0.5-0.9; p = 0.020) less chance of increasing the purchase of fresh foods and their physical activity, respectively.

**Figure 3:**
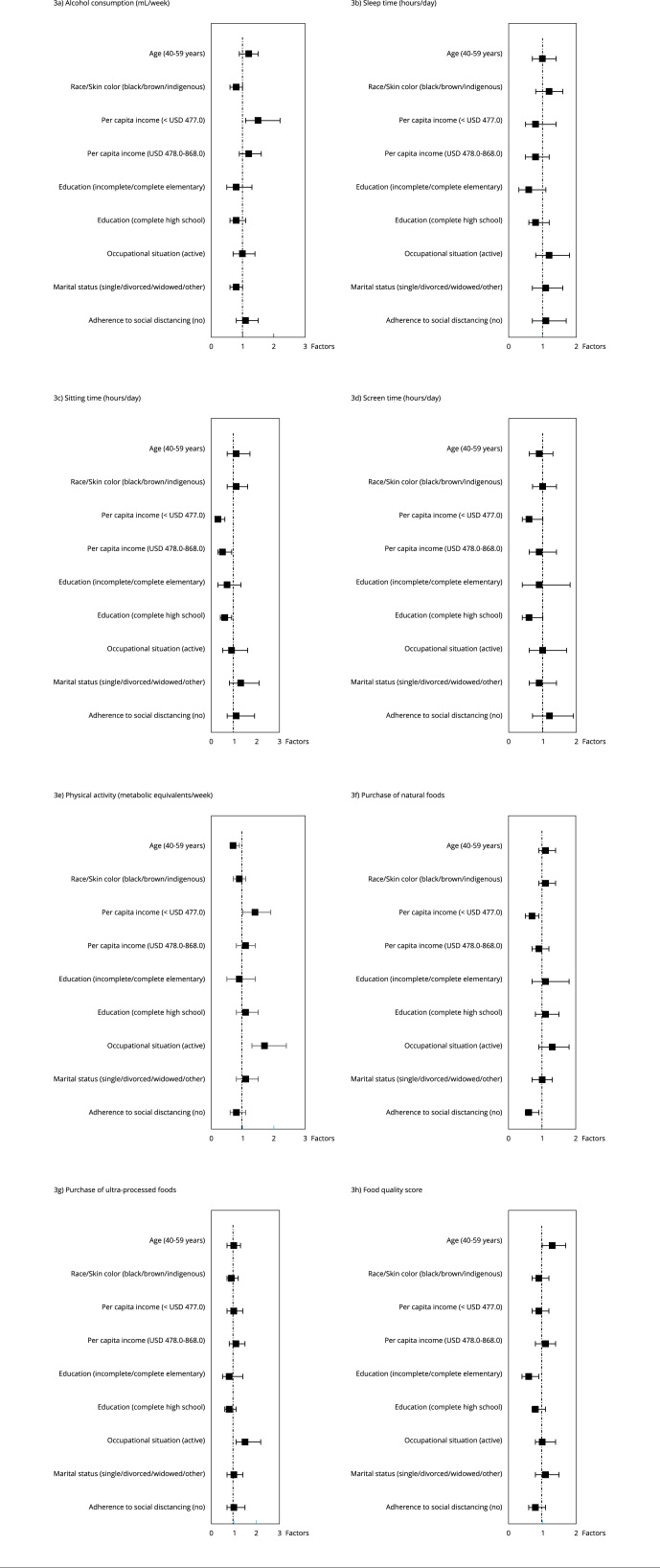
Factors associated with changes in lifestyle habits during the COVID-19 pandemic among men. *Brazilian Longitudinal Study of Adult Health* (ELSA-Brasil), 2017-2021.

Note: multinomial logistic regression on the dependent variables with three categories − reference to the first (decreased), category not shown. Binary logistic regression on dichotomous dependent variables. Final model adjusted for age, skin color, per capita income, education, occupational status, marital status and adherence to social distancing when p < 0.2 in the crude analysis. Data are odds ratios (95% confidence intervals). * p-value < 0.05; ** p-value < 0.001.

Regarding factors associated with changes in men’s lifestyle (Figure 3), it can be observed that individuals under 59 years of age were 30% more likely to increase the FQS (95%CI: 1.0-1.7; p = 0.020) and 30% less likely to increase physical activity (95%CI: 0.6-0.9; p = 0.004). Additionally, participants in the lowest income tertile were 30% (95%CI: 0.5-0.9; p = 0.020) less likely to increase the purchase of fresh foods and 70% (95%CI: 0.2-0.6; p = 0.001) less likely to increase sitting time, and this likelihood decreased to 50% (95%CI: 0.3-0.9; p = 0.020) as individuals’ income increased. The lowest income tertile was also a factor that increased the chance of alcohol consumption (OR = 1.5; 95%CI: 1.1-2.2; p = 0.010). Having the lowest level of education decreased by 40% (95%CI: 0.5-0.9; p = 0.020) the chance of increasing the FQS, and those with complete secondary education had 40% less chance of increasing screen time and sitting time (95%CI: 0.4-1.0; p = 0.040; and 95%CI: 0.4-0.9; p = 0.010, respectively).

Active male workers were 1.5 (95%CI: 1.1-2.2; p = 0.020) times more likely to increase the acquisition of ultra-processed foods, but they were also 1.7 (95%CI: 1.3-2.4; p = 0.001) times more likely to increase physical activity. Again, marital status did not influence changes in habits. Non-adherence to social distancing was a factor that reduced by 40% (95%CI: 0.5-0.9; p = 0.007) the chance of men increasing their consumption of fresh foods.

## Discussion

Significant changes were identified in the lifestyle of employees of public higher education institutions during the COVID-19, and some socioeconomic, occupational and behavioral factors were associated with changes in the diet quality, acquisition of ultra-oricessed foods and fresh foods, consumption of alcoholic beverages, physical activity, screen time, sitting time, and sleep time.

We found an improvement in diet quality in both sex, with a higher score in females. Individuals under 59 years were more likely to increase the FQS, and, among men, those with less education were less likely to increase the FQS. Similarly, a cross-sectional study with 6,325 adults carried out during the COVID-19 pandemic in five countries (Brazil, Argentina, Peru, Mexico, and Spain) ^3^ identified that Brazilians had a higher proportion of those who improved their food consumption compared to the previous period of the pandemic. There was a positive association in the adoption of healthier dietary changes among younger people (30-49 years, OR = 1.4; 95%CI: 1.1-1.7) and a negative association among participants with lower educational levels (OR = 0.70; 95%CI: 0.5-0.8). The sample of this study consists of a population with higher education, a factor associated with better diet quality ^23^.

Still in the context of eating habits, we identified an increase in the purchase of ultra-processed foods and fresh foods, in both genders. Male workers were more likely to purchase ultra-processed foods, while female workers were more likely to purchase fresh foods. However, women under 59 years were more likely to purchase both healthy and unhealthy foods. Individuals with lower income and education, and those who did not adhere to social distancing, were less likely to increase fresh foods acquisition.

The literature differs on this issue. Cross-sectional study on changes in lifestyles during COVID-19, carried out with more than 45,000 Brazilians (≥ 18 years), identified that the frequency of consumption of healthy foods decreased. The authors did not find significant differences in the consumption of these foods between age groups, but they identified an increase in the consumption of ultra-processed foods, mainly chocolates/cookies and sweets/pieces of pie ^5^. Another study carried out with 10,116 adult participants observed a significant increase in the consumption of healthy foods, regardless of the sociodemographic stratum, and stability in the consumption of unhealthy foods ^24^. The difference in the findings may be due to the time of data collection, given the occurrence of different waves of contagion - which occurred from early 2020 to late 2021 - and presented distinctions in the number of individuals undergoing social distancing, and, consequently, it may have impacted consumption and behavior routines. A review addressing articles from 2020 to March 2021 on the consumption of ultra-processed foods by Brazilians ^9^ identified an increase in the intake of these products, especially among individuals with less education.

Despite the particularity of the moment, this research found a trend in the factors that influence food choices. In previous studies ^23^ ^,^ ^25^ ^,^ ^26^, it was observed that markers of healthy and unhealthy foods are associated with sex and education, where men and individuals with less education tend to consume more unhealthy foods. And, regarding social restriction, we can assume that individuals who did not adhere to distancing did not have the same amount of time available for preparing meals ^27^, which may have impacted the purchase of unprocessed foods.

This study found a significant increase in alcohol consumption in both genders. Women with work activities and men with lower income were more likely to increase alcohol consumption. Data from the *ConVid Behavior Survey* ^4^ showed a 17.6% increase in alcohol consumption in the Brazilian population during social restrictions, with no sex-related differences, and a review that evaluated changes in habits during the pandemic identified a negative impact on alcohol consumption in different locations ^10^. This increase may be due to the fact that, in an atypical moment of social restrictions, and without fixed schedules, individuals may have increased domestic alcohol consumption ^28^.

Due to the increase in alcohol intake observed, it is noteworthy that ethanol consumption is associated with several diseases, such as cardiovascular diseases and some types of cancer, and alcohol consumption has short- and long-term effects on the body, with no safe intake, in addition to impairing the body’s defense against infectious agents ^29^ ^,^ ^30^.

We also identified a significant reduction in physical activity in both genders. Furthermore, active workers were more likely to increase leisure-time physical activity. A household survey, carried out with adults from two medium-sized municipalities in a Brazilian state, observed that the pandemic negatively influenced the practice of physical activity. The prevalence of physical inactivity was 58.7% (95%CI: 52.8-64.3) from October to December 2020, higher than the prevalence of physical inactivity before the pandemic (39.7%; 95%CI: 35.6-43.8), and individuals in remote work were less likely to be physically inactive ^5^, data similar to ours, although we did not analyze the occupational factor in a stratified way, considering remote work.

Malta et al. ^4^ identified a decrease in the proportion of individuals who practiced physical activity before the pandemic (from 30.1%; 95%CI: 28.9-31.5 to 12.0%; 95%CI: 11.1-12.9), and also observed that men maintained a higher level of physical activity during the pandemic compared to women. This demonstrates gender inequalities in housework and in the time available for self-care, which may have worsened during the period ^28^.

Also in this study, individuals who did not adhere to social distancing had a lower chance of increasing leisure-time physical activity. A study carried out in Ibero-American countries3 showed that most participants reported practicing physical activity at home (57.1%), which may indicate that, even with the decrease in physical activity in both genders, there may have been a greater concern with personal health for some individuals during this period.

Contrary to what previous studies have indicated (2018-2021) ^31^ ^,^ ^32^, the current analysis found a lower chance of practicing physical activity among adult men, compared to older people. This trend may have been reversed at that time due to fear of the disease among the population aged 60 years or over, given the high mortality from COVID-19 in this age group ^33^, and the specificity of our sample (higher education and income).

Our study verified an increase in sedentary behavior, indicated by screen time and sitting time, in both genders, with younger women having a greater chance of increasing sitting time. Data from the *ConVid Behavior Survey* survey ^4^ also identified an increase in sedentary behavior, of 1 hour and 45 minutes in television use and 1 hour and 30 minutes in computer or tablet use during the pandemic, in addition to a greater average time in front of screens being reported by younger adults. In a systematic review, Patterson et al. ^6^ found that sedentary behavior - even considering the levels of physical activity practiced by individuals - is associated with chronic outcomes, of which, above 6 to 8 hours a day of sitting time increases the risk of mortality from all causes and cardiovascular diseases.

Among the factors that reduced the chance of screen time and sitting time in our study, is the fact that women work. Thus, it is clear that women’s double workload resulted in less time for sedentary behavior, especially in a period of social restrictions in which housework increased. According to ELSA-Brasil’s “COVID-19 Scenarios” ^28^, there was a threefold increase in domestic work among participants in remote work, and awork overload for women, with four hours more in housework than men. Lower income and education level were also preponderant factors in the lower chance of increasing screen time and sitting time, an expected result, since a study carried out with adults from a public educational institution in the State of Espírito Santo found that participants with higher education had higher means of sceen time and sitting time ^34^.

In addition to the aforementioned behaviors, we also identified a significant increase in the average sleep time in both genders, where women with complete secondary education were less likely to increase this time. An observational study carried out with the Brazilian adult population, which identified changes in lifestyle habits during the pandemic, found a significant increase in the participants’ sleep time ^35^. Other studies found a relationship between the duration and quality of sleep with females and lower education ^36^ ^,^ ^37^. However, a study with data from ISACamp 2014/2015 ^38^ (Campinas, São Paulo State), carried out with 1,969 adults, identified a greater chance of short sleep duration among men and those with higher education. The importance of maintaining a favorable sleep condition is emphasized, since sleep disorders and deprivation are related to cardiometabolic diseases and obesity ^39^ ^,^ ^40^.

Social restrictions during the COVID-19 pandemic contributed to changing the habits of individuals in various locations worldwide, given the difficulty in maintaining healthy lifestyles, leading, in some cases, to inappropriate behaviors such as physical inactivity, sedentary behavior, consumption of ultra-processed foods and alcoholic beverages, which, if continued, may lead to insulin resistance, abdominal obesity, inflammatory markers, immune functions, and cardiometabolic diseases, with an impact on public health ^2^ ^,^ ^7^.

We must mention the limitations of this study, such as the fact that the participants answered an online questionnaire about the wave-COVID, which may represent information or measurement bias. However, this was the strategy used to reach the participants without exposing them to COVID-19. Some questions are not validated. Furthermore, the results cannot be extrapolated to the general population. It is worth mentioning that ELSA-Brasil recommends standards that ensure data quality, and the study used information from participants in two different collection stages, thus providing more accurate results.

The results found provide knowledge about individual habits that impact chronic noncommunicable events, especially among those with greater risk factors, and, consequently, impact public health. By understanding the behaviors generated by a health crisis within the territory, it is possible to formulate policies and schedule public interventions in periods similar to the COVID-19 pandemic, to encourage healthy behaviors. It is worth emphasizing the need to find out whether the changes in the participants’ lifestyles were sustained over time, following the “normalization” of routines with the immunization and, later, with the control of COVID-19. It is important to assess the impact of these changes on the individuals’ health.

We provide answers regarding the role of socioeconomic, occupational and social distancing factors in changing habits that are more harmful to health. Age and active work were points that contributed to increasing the chance of ultra-processed foods acquisition, and age and adherence to social distancing influenced the change to a more sedentary behavior, while income and active work favored an increase in alcohol consumption. Therefore, such factors should be considered when developing public policies, in order to avoid individual behaviors that are related to the weakening of the immune system, the occurrence of chronic diseases and other health implications.
