# Supplementary material for: Impact of socioeconomic factors on lifestyle changes among employees of public higher education institutions in ELSA-Brasil during COVID-19 pandemic
Source: Cad Saude Publica. 2023 Nov 13;39(11):e00047123. doi: 10.1590/0102-311XEN047123 (PMC10644976; doi:10.1590/0102-311XEN047123)
Supplement: Supplementary file 1 [file 1678-4464-csp-39-11-EN047123-s.pdf]

Supplementary Material

Table S1 *Brazilian Longitudinal Study Adult Health* (ELSA-Brasil) food quality score.

Foods/Food Groups	Score according to frequency of consumption				
	1 or + x/day	5-6 x/week	1-4x/week	1-3x/ month	Never or almost never
Homebread/Roll bread	0	0	0	0	0
Industrialized bread (loaf, light, whole grain bread..)	-1	-1	0	+1	+1
Rice/oats/Corn/Other cereals	0	0	0	0	0
Potato/Yam/Cassava/Other tubers	0	0	0	0	0
Fruits	+1	0	-1	-1	-1
Vegetables	+1	0	-1	-1	-1
Legumes (beans, chickpeas, lentils)	+1	+1	0	-1	-1
Fish	+1	+1	+1	-1	-1
Dried fruits (Brazil nuts, walnuts, almonds, hazelnuts)	0	0	0	0	0
Egg (fried, boiled, scrambled, poached, omelet)	0	0	0	0	0
Milk (whole, semi-skimmed, skimmed, or powdered)	0	0	0	0	0
Industrialized yogurt	-1	-1	0	+1	+1
White cheeses (raw cheese, ricotta, cottage, buffalo mozzarella)	0	0	0	0	0
Cheeses (mozzarella, cheddar, canasta, processed polenghi type)	-1	-1	0	+1	+1
Butter/Margarine	-1	-1	0	+1	+1
Meat (beef, pork, chicken)	-1	-1	+1	+1	+1
Processed meat (sausages, ham or others)	-1	-1	0	+1	+1
Cookies (sweet, salty cracker, with or without filling)	-1	-1	0	+1	+1
Fast food/frozen food (lasagna, pizza, burger..)	-1	-1	0	+1	+1
Savory snacks (snacks and fried foods in general)	-1	-1	0	+1	+1
Sweets (Ice cream, chocolate, pudding, cakes, gelatin..)	-1	-1	0	+1	+1
Soft drinks and boxed juices	-1	-1	0	+1	+1
